# Generation of Adaptive Immune Responses Following Influenza Virus Challenge is Not Compromised by Pre-Treatment with the TLR-2 Agonist Pam_2_Cys

**DOI:** 10.3389/fimmu.2015.00290

**Published:** 2015-06-05

**Authors:** Edin Jessica Mifsud, Amabel C. L. Tan, Lorena Elizabeth Brown, Brendon Yew Loong Chua, David C. Jackson

**Affiliations:** ^1^Department of Microbiology and Immunology, Peter Doherty Institute for Infection and Immunity, The University of Melbourne, Parkville, VIC, Australia

**Keywords:** Pam_2_Cys, toll-like receptor-2, influenza A virus, innate immunity, adaptive immunity

## Abstract

Immunostimulatory agents provide a new category of anti-microbial agents that activate the host’s innate immune system allowing control of viral and/or bacterial infections. The TLR-2 agonist PEG-Pam_2_Cys has been shown to mediate potent anti-viral activity against influenza viruses when administered prophylactically (1). Here, we demonstrate that the treatment of mice with PEG-Pam_2_Cys does not compromise their ability to generate adaptive immune responses following subsequent challenge with influenza virus. The antibody induced in mice pre-treated with Pam_2_Cys possessed hemagglutination-inhibiting activities and the CD8^+^ T-cell responses that were elicited provided protection against heterologous viral challenge. In the absence of an effective influenza vaccine, an agent that provides immediate protection against the virus and does not compromise the induction of influenza-specific immunity on exposure to infectious virus provides an opportunity for population immunity to be achieved through natural exposure to virus.

## Introduction

Vaccination is currently the most effective medical intervention available for prevention of influenza infection and disease. The efficacy of influenza vaccines is dependent on a match between the viral strains included in the vaccine and circulating influenza virus strains. Although anti-viral drugs can diminish symptoms and shorten the duration of illness (2, 3), the suggestion has been made that in the case of influenza their use can hamper the development of immunological memory (4) leaving the host susceptible to re-infection when drug treatment ceases. Although anti-influenza drugs continue to be used effectively in a clinical setting, concerns with anti-viral resistance (5–7) due to their widespread use have resulted in increased efforts to develop alternative prevention strategies.

One approach is to harness the rapid anti-microbial responses of the innate immune system, particularly for pathogens that use the respiratory portal of entry. Immunostimulatory agents provide immediate non-specific protection against virulent influenza challenge (1, 8–10), and in mice, these compounds reduce the symptoms associated with influenza infection and also the viral burden reducing morbidity and promoting survival. Furthermore, by acting through the innate immune system and not directly on the pathogen, these immunostimulatory agents are unlikely to select resistant virus variants.

Many immunostimulatory agents are agonists of toll-like receptors (TLR) and mediate anti-viral and anti-bacterial activity by activating inflammatory pathways (1, 8–10). An understanding of the way in which these compounds affect acquisition of adaptive immunity and immunological memory is important because they could be employed for use during influenza pandemics where there is a high risk of re-exposure to pathogen. In the case of respiratory diseases, delivery of TLR agonists to the pulmonary tract can alter the immune environment, which could influence subsequent induction of adaptive immunity. An understanding of the way in which immunostimulatory agents, including TLR agonists, affect development of pathogen-specific immunity is therefore important.

We have developed a soluble form of the synthetic analog, *S*-[2,3-bis(palmitoyloxy)propyl] cysteine (Pam_2_Cys), which acts as an agonist of toll-like receptor-2 (TLR-2) and provides immediate protection against challenge with a lethal dose of influenza virus A/PR/8/34 (PR8, H1N1) (1). We have also shown that although Pam_2_Cys treatment significantly reduces viral burden, reduces disease symptoms, and prevents death, it does not totally abrogate infection. This property provides the potential to develop immunity to influenza virus through asymptomatic natural infection.

In this study, we show that Pam_2_Cys prophylaxis permits development of a robust influenza virus-specific adaptive immune responses comprised CD8^+^ T-cells, CD4^+^ T-cells, and antibodies. We also show that this virus-independent stimulation of the innate immune system does not compromise the development of heterologous immunity. The development of an agent that can provide the host with immediate protection and does not hinder the development of pathogen-specific immunity following exposure to infectious virus provides an opportunity for population immunity to be achieved through natural exposure to the virus.

## Materials and Methods

### Synthesis of PEG-Pam_2_Cys

Pam_2_Cys is hydrophobic and insoluble in physiological media; therefore, we synthesized the agonist with a polyethylene glycol molecule attached to confer solubility and allow administration by the intranasal (i.n.) route. PEG-Pam_2_Cys was synthesized in house using Fmoc-based chemistry as described previously (1). The product was purified by reversed-phase high-performance liquid chromatography (HPLC) using a C4 VYDAC column (10 mm × 250 mm; Alltech, NSW, Australia) installed in a Waters HPLC system (Waters Millipore, Milford, MA, USA). The purity of PEG-Pam_2_Cys was determined by HPLC using a VYDAC C8 column (4.6 mm × 250 mm) installed in a Waters system and was found to be >95%. The authenticity of the product was determined by mass analysis (mass value found 1,502.2 Da; expected mass 1,502.1 Da) using an Agilent 1100 Series LC/MSD ion-trap mass spectrometer (Agilent, Palo Alto, CA, USA).

### Animals

Female C57BL/6 mice (6–12 weeks old) were bred and housed in the Animal house facility at the Department of Microbiology and Immunology, The University of Melbourne. All animal experiments were conducted with approval from the University of Melbourne Animal Ethics Committee.

### Inoculations and viral challenge

Mice were anesthetized using isoflurane inhalation and received either 20 nmol of PEG-Pam_2_Cys dissolved in saline or saline alone by the i.n. route.

Mice were challenged with a virulent strain of influenza virus 3 days after treatment with PEG-Pam_2_Cys. 200 plaque forming units (PFU) of the H1N1 influenza virus A/Puerto Rico/8/34 (PR8, Mount Sinai) were administered by the i.n. route during isoflurane anesthesia. Infection following challenge with PR8 in this way results in weight loss, labored breathing, and a hunched posture; animals were monitored daily for any signs of illness and euthanized at a pre-determined humane endpoint.

Challenge with less virulent strains of influenza virus were also carried out during isoflurane anesthesia with either (i) 10^4.5^ PFU of the H3N1 virus Mem/Bel (a genetic reassortant of A/Memphis/1/71 [H3N2] × A/Bellamy/42 [H1N1]) (ii) 10^4.5^ PFU of Mem71 A/Memphis/1/71, an H3N2 strain or (iii) 10^4.5^ PFU of the H3N2 virus, ×31. Each virus preparation was diluted in PBS and administered i.n. 3 days after receiving PEG-Pam_2_Cys.

### Preparation of lung cells

Following CO_2_ asphyxiation, lungs were removed and subjected to enzymatic digestion with collagenase A (2 mg/ml; Roche, Mannheim, Germany) in RPMI 1640 medium for 30 minutes 37°C. Cells were strained through a metal sieve and treated with ammonium-Tris hydrochloride (7.4% w/v ammonium chloride, 2.06% w/v Tris hydrochloride [ATC]) for 5 min at 37°C and were then washed twice with RP-10 (RPMI 1640 containing 10% fetal calf serum, 2 mM l-glutamine, 76 mM 2-meceptoethanol, 150 U/ml penicillin, 150 mg/ml streptomycin, 150 mM non-essential amino acids [all supplements were obtained from Life Technologies]) in 7.5 mM HEPES. The number of viable cells was determined using trypan blue exclusion.

### Intracellular cytokine staining

Lung and spleen cells (2–3 × 10^6^/200 μl) were stimulated in the presence or absence of peptide PA _224–236_ or NP_366–374_ (1 μg/ml) with 5 μg/ml of GolgiPlug (BD Biosciences Pharmingen) 25 U/ml recombinant human IL-2 (Roche, Indianapolis, IN, USA) for 6 h at 37°C. Following stimulation, cells were stained with PercP5.5 anti-mouse CD8α (BD Biosciences Pharmingen) then fixed and permeabilized using the BD Cytofix/Cytoperm kit (BD Biosciences Pharmingen) according to the manufacturer’s instructions. Cells were finally stained with FITC-labeled antibody directed against interferon-γ (IFN-γ), Pe-Cy7-labeled antibodies directed against tumor necrosis factor-α (TNF-α) and APC-labeled antibodies directed against IL-2. Samples were analyzed using a FACSCanto II and analyzed using FlowJo Software.

### Hemagglutination inhibition assay

Sera were prepared from blood and stored at −20°C until use. To remove any non-specific inhibitors of hemagglutination, sera were diluted ^1^/_5_ with receptor destroying enzyme (RDE II, Denka Seiken Co., Ltd.) and incubated at 37°C overnight. Sodium citrate (1.6% w/v from Merck; Kilsyth, Victoria) diluted in PBS (Media Preparation Facility, Department of Microbiology and Immunology). 0.1% sodium citrate (Chem Supply) was added and samples were incubated for a further 2 h at 56°C prior to use. The hemagglutination inhibition (HI) assay was performed using either Mem/Bel or PR8 according to the method described in Ref. (11) and modified to a micro-titer format.

### *In vivo* cytotoxic T-cell assay

An *in vivo* cytotoxic T-cell (CTL) assay was performed in mice that had been primed with Mem71 virus and challenged 1 month later with PR8 virus using a previously described method (12). The data generated were analyzed using FlowJo software and the percentage specific lysis of CFSE-labeled target cells in each mouse calculated using the following equation:

$$\%\text{specific lysis}=1-\frac{r\left(\text{non-infected}\right)}{r\left(\text{infected}\right)}*100$$

$$\text{where r}=\%\frac{\text{CFSE low}}{\text{CFSE high}}$$

### CD4^+^ T-cell IFN-γ detecting ELISPOT

Membrane-based 96-well plates (MAIPS4510; Millipore, North Ryde, NSW, Australia) were coated with anti-mouse IFN-γ capture antibody (clone R4-6A2; Pharmingen) prior to addition of 5 × 10^5^ cells to each well followed by 50 μl NP_311–325_ peptide (5 μg/well). Four wells lacking peptide were included as negative controls. Cells were cultured for 18 h at 37°C 5% CO_2_ and IFN-γ detected using biotinylated mouse anti-IFN-γ detection Ab (clone XMG1.2; Pharmingen) and streptavidin–alkaline phosphatase (Pharmingen) as described elsewhere (13). Spots formed by the deposition of enzyme substrate were counted using an ELISPOT plate reader (AID Autoimmun Diagnotika, Strassberg, Germany) and analyzed using AID software. The number of spot-forming units (SFU) was calculated by subtracting the sum of the background value plus two SD and responses considered positive when the net SFU value was >20 SFU/10^6^ cells.

### Determination of influenza virus titers

Lung viral titers were determined using an MDCK cell-based plaque assay as previously described (14).

### Characterization of the pulmonary cytokine environment

The levels of IFN-γ, TNF-α, interlukin-6 (IL-6), IL-10, IL-12p70, and monocyte chemoattractant protein-1 (MCP-1) in bronchoalveolar lavage (BAL) fluid or lung tissue were analyzed using a BD Cytometric Bead Array (CBA) Mouse Inflammation Kit (BD Biosciences, San Diego, CA, USA) according to the manufacturer’s instructions with the exception that a total of 2 μl of each capture bead was used in 50 μl of BAL sample and the PE-detection reagent was diluted 1 in 5. Samples were analyzed using a Becton Dickinson FACSCalibur flow cytometer and data analyzed using the FlowJo software package (Tree Star, Inc., Ashland, OR, USA).

### Statistical analyses

For comparison of two data sets, a two-tailed Student’s *t*-test was used. For comparison of data sets with a non-Gaussian distribution, a Mann–Whitney *t*-test was used. A *P-*value ≤ 0.05 was considered statistically significant. Statistical analyses were carried out using the GraphPad Prism 6 software package.

## Results

### Adaptive immune responses generated following challenge with virulent and non-virulent influenza virus strains

In the C57BL/6 mouse model of influenza infection, the neutralizing Ab response is directed predominantly to the viral hemagglutinin and the CD8^+^ T-cell response is directed to the two immunodominant epitopes PA_224–236_ and NP_366–374_, present on the internal proteins, acid polymerase and nucleoprotein, respectively (15).

**Figure 1:**
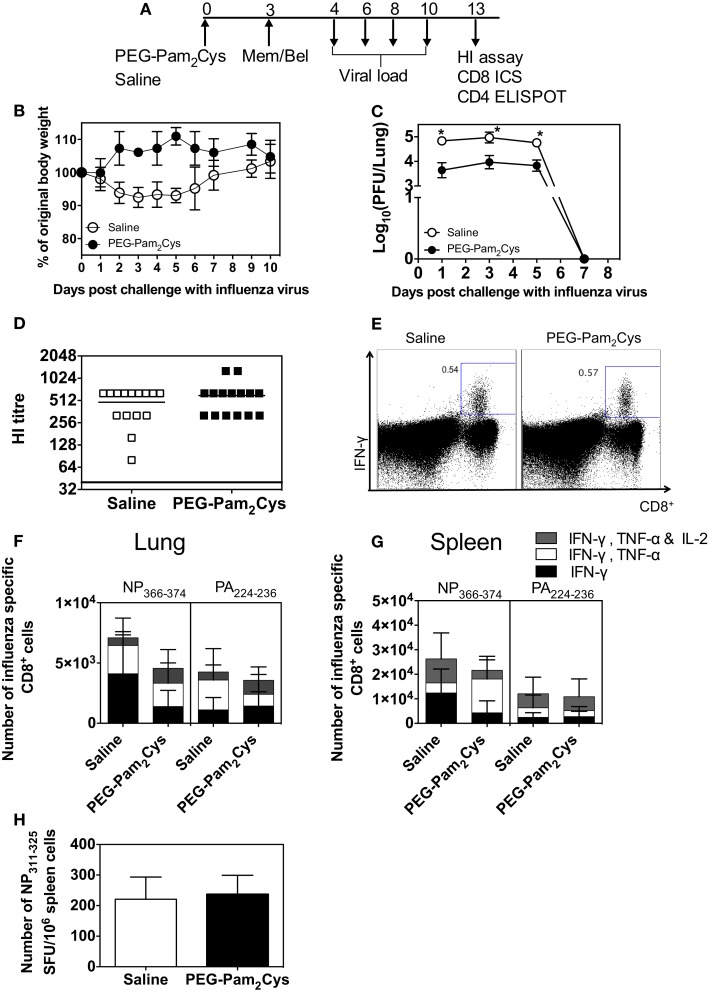
**Antibody and cell mediated immune responses generated in PEG-Pam_2_Cys-treated mice following infection with Mem/Bel**. **(A)** Timeline of protocols used; C57BL/6 (*n* = 5–15) mice were inoculated via the i.n. route with saline or 20 nmol of PEG-Pam_2_Cys 3 days prior to challenge with 10^4.5^ PFU of Mem/Bel. At various times following challenge, mice were culled and various virological and immunological parameters measured. **(B)** Percentage weight change following influenza challenge. **(C)** Mean viral loads present in the lung throughout the course of infection. **(D)** Influenza-specific Ab levels in sera were determined using HI assays. **(E)** Representative FACS plots of the NP_366–374_-specific response in the lungs of PEG-Pam_2_Cys and saline-treated mice. Numbers of NP_366–374_ and PA_224–236_ specific cytokine secreting CD8^+^ T-cells in **(F)** lungs and **(G)** spleen. **(H)** Number of IFN-γ secreting CD4^+^ T-cells [spot-forming units (SFU) per 10^6^ cells] in spleen cells following stimulation with peptide epitope NP_311–325_. Statistical significance for **(B)** was determined using an unpaired Mann–Whitney *t*-test (*P < 0.05).

Use of non-virulent Mem–Bel (H3N1) influenza virus allowed us to follow the adaptive immune response beyond 7 days, the time point at which mice infected with virulent PR8 (H1N1) influenza virus succumb to infection. Saline or PEG-Pam_2_Cys was administered to mice 3 days prior to challenge with 10^4.5^ PFU of Mem–Bel virus (Figure 1A), mice were monitored daily following influenza challenge and weight loss is shown in Figure 1B. Animals that received PEG-Pam_2_Cys maintained their overall bodyweight throughout the duration of infection, whereas animals treated with saline lost a small amount of weight during influenza challenge but regained weight 10 days after influenza challenge. The viral load in mice treated with PEG-Pam_2_Cys was significantly reduced (>1 log) during the early stages of infection and by day 7, virus was cleared from the lungs of both treatment groups (Figure 1C).

No significant differences in titers of HI antibodies (*P* = 0.4977) were detected in the sera of saline and PEG-Pam_2_Cys-treated animals 10 days after challenge (Figure 1D). Epitope-specific CD8^+^ T-cell responses were examined 10 days postinfection, and no significant differences were detected in cytokine secreting CD8^+^ T-cells present in the lungs or spleens regardless of the treatment received (Figures 1E–G). Gating strategy used to identify cytokine secreting cells is shown in Figure S1 in Supplementary Material. We also detected no significant (*P* = 0.7381) numbers of CD4^+^ T-cells in the spleens of each treatment group (Figure 1H). Taken together, the results suggest that neither cell-mediated nor humoral immune responses were compromised by pre-treatment with PEG-Pam_2_Cys.

**Figure 2:**
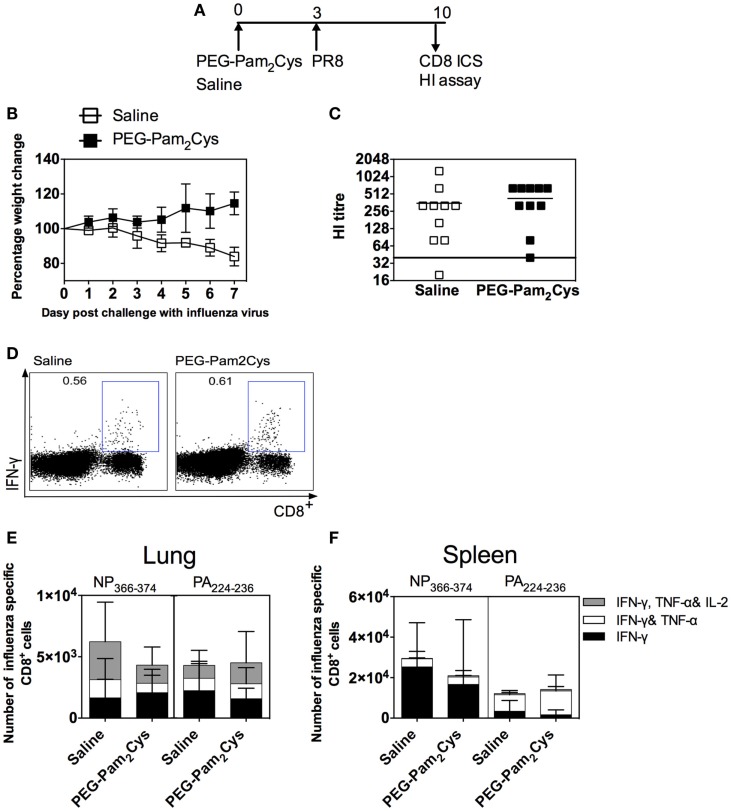
**Antibody and cell mediated immune responses generated in PEG-Pam_2_Cys-treated mice following challenge with 200 PFU of PR8 influenza virus**. **(A)** Timeline of protocols used; C57BL/6 (*n* = 5–10) mice were inoculated via the i.n. route with saline or 20 nmol of PEG-Pam_2_Cys 3 days prior to challenge with 200PFU of PR8. **(B)** Mice were monitored daily following viral challenge and the percentage change in body weight recorded. **(C)** Hemagglutination inhibiting Ab levels in sera. **(D)** Representative FACS plots of the NP_366-374_-specific CD8^+^ T-cell response in lungs. Numbers of NP_366–374_ or PA_224–236_ specific cytokine secreting CD8^+^ T-cells in **(E)** lungs and **(F)** spleen. Statistical significance was determined using an unpaired Student’s *t*-test (*P < 0.05).

To determine the effects of PEG-Pam_2_Cys on infection with a more virulent influenza virus, mice were treated with saline or the immunostimulant and subsequently challenged with a lethal dose of PR8 virus (Figure 2A). Saline control mice suffered substantial weight loss and reached the previously determined humane end point 8 days after challenge (Figure 2B). In contrast, mice pre-treated with PEG-Pam_2_Cys all survived viral challenge, a result which is consistent with our previous findings (1).

Another group of C57BL/6 mice treated with either saline or PEG-Pam_2_Cys and subsequently challenged with PR8 were euthanized 7 days after viral challenge to asses the adaptive immune responses. Non-significant titers of HI antibodies (*P* = 0.2607) were detected in sera of animals 7 days after challenge with PR8 virus (Figure 2C) whether they had been pre-treated with saline. When the fine specificity of the CD8^+^ T-cell response was examined, very few differences were detected in the cytokine profiles of PA_224–236_ and NP_366–374_-specific CD8^+^ T-cells obtained from lungs and spleen of mice whether treated with saline or PEG-Pam_2_Cys (Figures 2D–F). The results again indicate that treatment with PEG-Pam_2_Cys has little or no deleterious effect on the development of adaptive immune responses.

### PEG-Pam_2_Cys treatment does not affect the development of recall CD8 T-cell responses or the development of heterologous immunity

Because CD8^+^ T-cells target the internal, conserved epitopes of the influenza virus proteins, they are able to recognize a broad range of influenza strains (16). These cells are, however, short lived and require constant stimulation for persistence. The use of Mem’71 (H3N2) results in a resolving infection, which allowed us to track the maintenance of influenza-specific immune responses and also to determine the ability to provide protection against a second challenge with a heterologous strain of virus.

**Figure 3:**
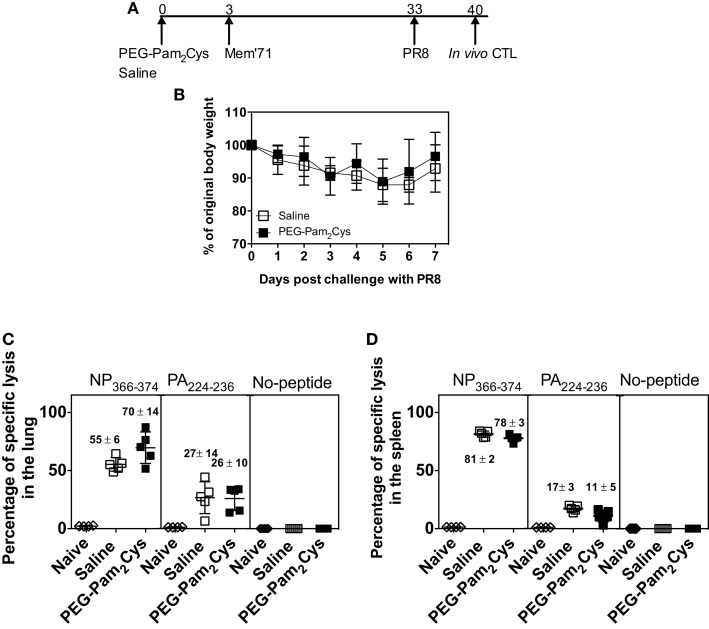
**Influenza-specific cytotoxic CD8^+^ T-cells persist in the lung and the spleen of PEG-Pam_2_Cys-treated mice**. **(A)** Time line of protocol used; C57BL/6 mice (*n* = 5) received saline or PEG-Pam_2_Cys 3 days prior to challenge with 10^4.5^ PFU of Mem71 influenza virus. One month later, mice were challenged with a lethal dose of PR8. **(B)** Percentage weight change after secondary influenza challenge. Seven days after challenge with PR8 naïve “donor” splenic cells were differentially labeled with CFSE and pulsed with no peptide, peptide NP_366–374_, or peptide PA_224–236_ before intravenous transfer via the base of tail into recipient mice. Recipient mice were killed and remaining labeled donor cells in the lungs and spleens enumerated using flow cytometry. The percentage of specific lysis observed in the lung **(C)** and spleen **(D)** are shown. Each symbol in **(C,D)** represents the percentage of specific lysis obtained by individuals and the vertical line indicates the mean of each group. Numbers above each group indicate the mean amount of specific lysis of each groups with the SD. Data are from one of the two independent experiments, which yielded similar results.

To examine the long-term functional and cross-protective capabilities of the CD8^+^ T-cell response generated, we assessed CD8^+^ T-cell responses using an *in vivo* CTL assay. Following treatment with PEG-Pam_2_Cys or saline and subsequent challenge with Mem’71 (H3N2) virus, mice were then challenged 4 weeks later with a lethal dose of the heterologous PR8 (H1N1) virus (Figure 3A). The results (Figure 3B) demonstrate that both groups were protected from lethal PR8 challenge, which typically causes 20% weight loss by day 7 (Figure 2B), indicating that treatment with Pam2Cys does not compromise the ability to elicit and maintain immunity against heterologous virus challenge.

Seven days after secondary infection splenocytes from naïve, “donor” mice were pulsed with either PA_224–236_ peptide, NP_366–374_ peptide or received no treatment. The cells were then differentially labeled with different concentrations of CFSE and injected intravenously via the base of tail into recipient mice. After 14 h, labeled cells present in lungs and spleen were enumerated by flow cytometry and the gating strategy is shown in Figure S2 in Supplementary Material. The difference in the number of CFSE-labeled cells in infected mice compared to uninfected mice revealed that the CD8^+^ T-cell response generated in mice pre-treated with PEG-Pam_2_Cys or saline were equally effective at killing donor cells (Figures 3C,D). The results clearly demonstrate that prophylaxis with PEG-Pam_2_Cys did not compromise the function or quality of the CD8^+^ T-cell response generated. The results of the experiments further demonstrate that the immunostimulatory effects of PEG-Pam_2_Cys do not affect the cytotoxic capabilities of T-cells responsible for influenza-specific immunity.

**Figure 4:**
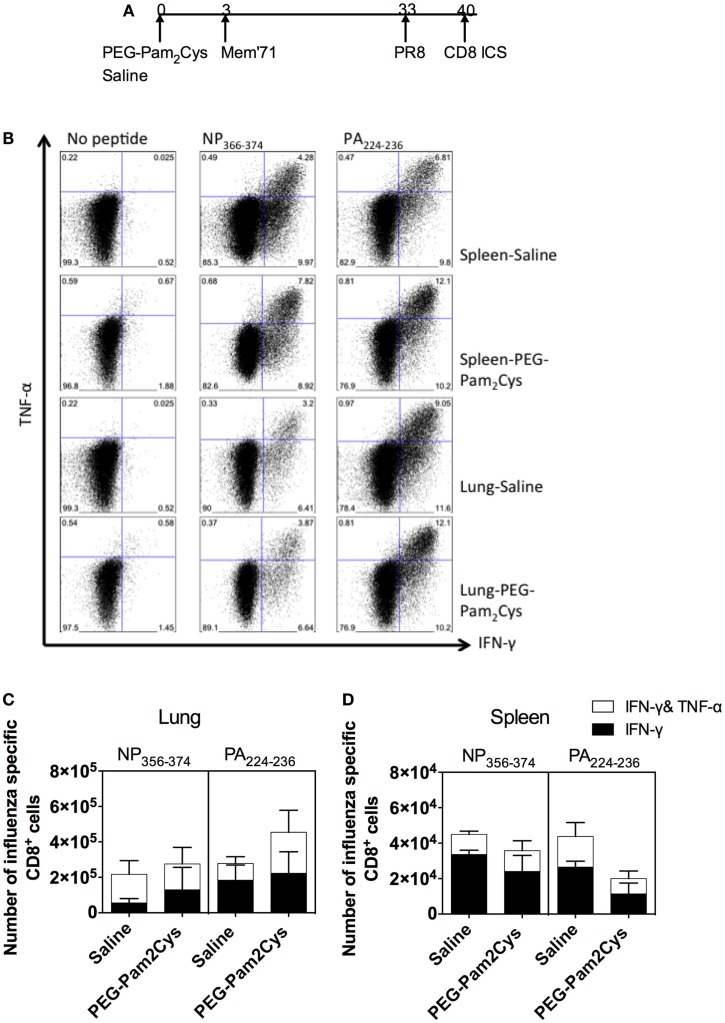
**Influenza-specific CD8^+^ T-cell responses persist in the spleen and lung following stimulation with PEG-Pam_2_Cys**. **(A)** Timeline of protocol used; C57BL/6 mice (*n* = 5) received 20 nmol of PEG-Pam_2_Cys or saline 3 days prior to challenge with 10^4.5^ PFU of Mem71. One month after primary challenge, mice were challenged with PR8 influenza virus, and 7 days later, an ICS assay was performed to examine the cytokine profile of influenza-specific CD8^+^ T-cells that were generated. **(B)** Representative FACS plots show the percentage of CD8^+^ T-cells from the spleen secreting either IFN-γ and/or TNF-α. Numbers of NP_366–374_ or PA_224–236_ specific CD8^+^ T-cells secreting cytokines in lung **(C)** and spleen **(D)**. Results are expressed as the mean (±1 SD). Data are from one of the two independent experiments that yielded similar results.

To further characterize the CD8^+^ T-cell response, the cellular cytokine profiles were examined by ICS (Figure 4A) and the gating strategy is shown in Figure S3 in Supplementary Material. There were no significant differences in the numbers of PA_224–236_ or NP_366–374_-specific T-cells capable of secreting a combination of cytokines in the lungs and spleens of saline and PEG-Pam_2_Cys treatment groups (Figures 4B–D). These results confirm our earlier findings (1) that Pam2Cys does not hinder development of influenza-specific immune responses. We now show that the influenza-specific immune response can be recalled by secondary infection with a different influenza virus and that these cells possess cytolytic function and secrete a combination of cytokines associated with protection.

### Pam_2_Cys treatment does not alter adaptive immune responses generated in immunologically experienced mice

**Figure 5:**
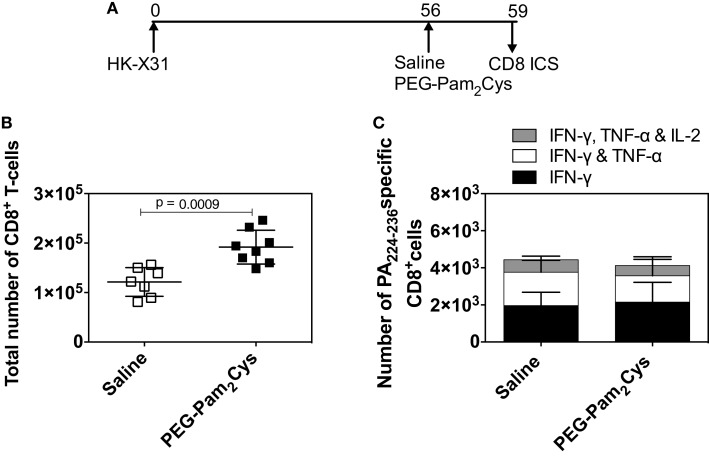
**PEG-Pam_2_Cys treatment alone does not activate memory CD8^+^T-cells**. **(A)** Timeline of protocol used; C57BL/6 mice were challenged with 10^4.5^ PFU of X31 virus, and 2 months later, mice received either saline or 20 nmol of PEG-Pam_2_Cys. After 3 days, mice were euthanized and the CD8^+^ T-cell responses assessed. **(B)** Total number of CD8^+^ T-cells in the lung. **(C)** Numbers of PA_224–236_ specific CD8^+^ T-cells secreting IFN-γ, IFN-γ, and TNF-α or IFN-γ, TNF-α, and IL-2 in the lung. Results are expressed as the mean (±1 SD). Statistical significance is denoted * on the graph and was determined using an unpaired Student’s *t*-test (*P* < 0.05).

Following vaccination or natural infection, human beings are no longer immunologically naïve. If immunostimulatory agents are to be used in human beings, we need to determine whether or not they affect existing antigen specific T-cells. Others (17, 18) have shown that subsequent and heterologous influenza virus infections cause an influx of CD8^+^ T-cells into lungs. These infections, or more specifically the inflammation that they induce, can lead to the recruitment of cells into the lung (19). What we have previously observed following treatment of immunologically naïve animals with PEG-Pam_2_Cys (1) is an increase in the numbers of CD8^+^ T-cells. We therefore determined whether or not PEG-Pam_2_Cys delivered intanasally affected resident CD8^+^ T-cells elicited by previous infection. Immunologically experienced mice were generated by challenge with a non-lethal dose of X31 influenza virus and 2 months later, mice were treated with saline or PEG-Pam_2_Cys (Figure 5A). We observed an increase in the number of CD8^+^ T-cells in lungs of mice treated with PEG-Pam_2_Cys (Figure 5B) supporting our earlier observations (1) but did not observe significant differences in the number and cytokine profiles of PA_224–236_ specific CD8^+^ T-cells (Figure 5C) suggesting that Pam_2_Cys treatment does not activate memory CD8^+^ T-cells.

**Figure 6:**
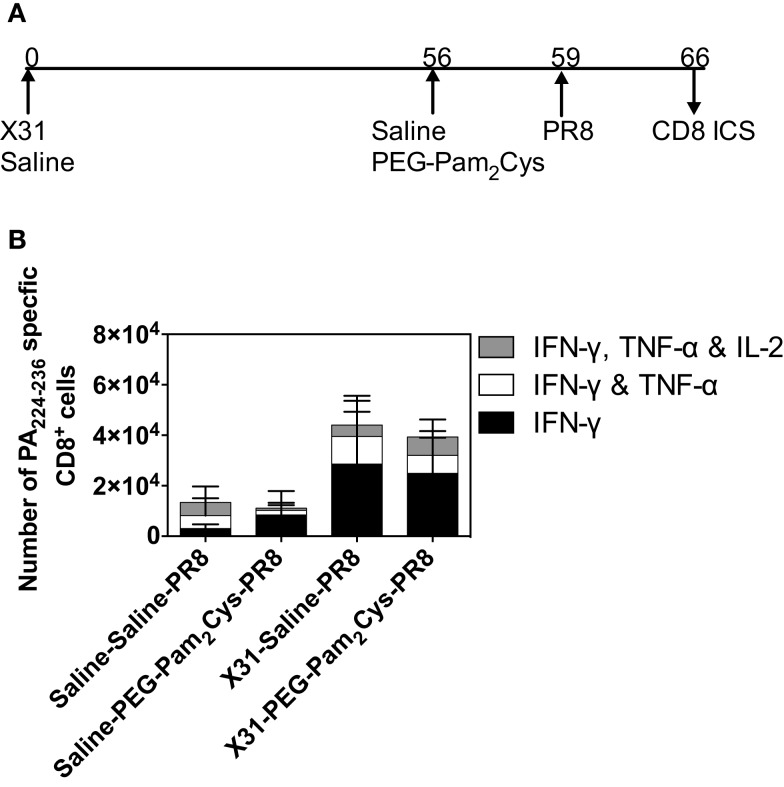
**Pre-existing adaptive immune responses are not compromised by stimulation with Pam_2_Cys**. **(A)** Timeline of infection protocol used; C57BL/6 mice (*n* = 5) were challenged with 10^4.5^ PFU of ×31 virus, and 2 months later, mice received either saline or 20 nmol of PEG-Pam_2_ Cys 3 days prior to challenge with a lethal dose of PR8 virus. Mice were monitored daily following challenge and the PA_224–236_ specific CD8^+^ T-cells secreting cytokine in the lung are shown in **(B)**. Results are expressed as the mean (±1 SD).

We next determined whether PEG-Pam_2_Cys altered the ability of mice to recall a previous immune state. Using the treatment regime shown in Figure 6A, the PA_224–236_ specific CD8^+^ T-cell response was examined 7 days after challenge with PR8 virus. Comparable numbers of CD8^+^ T-cells secreting IFN-γ alone, IFN-γ plus TNF-α, or IFN-γ plus TNF-α plus IL-2 were detected in the lungs of mice (Figure 6B). Taken together, the data suggest that stimulation of the innate immune system with PEG-Pam_2_Cys does not impact secondary recall responses.

## Discussion

In this study, we have shown that PEG-Pam_2_Cys treatment provides the host with immediate defense against influenza by reducing viral burden, eliminating disease symptoms, and also allows the generation of adaptive immune responses that are quantitatively and qualitatively similar to those generated during natural infection.

The cellular and humoral arms of the adaptive immune system each combat influenza virus infection and both mitigate disease severity. It is therefore necessary that immunomodulatory agents developed to target influenza should not hinder the development of either arm of the adaptive immune response. Even though PEG-Pam_2_Cys treatment causes a dramatic reduction (~90%) in antigen load compared with treatment with saline, similar numbers of CD8^+^ T-cells were elicited. This is unexpected given previous findings (20–22) that decreased antigen loads have profound effects on resulting T-cell responses. Possible explanations for this are the enhanced proliferation of CD8^+^ T-cells, due to their expression of TLR-2, following stimulation with TLR-2 ligands even in the absence of co-stimulation by APCs (23). Direct activation of TLR-2 has also been shown to reduce the amount of antigen required for CD8^+^ T-cell activation even promoting proliferation of CD8^+^ T-cells with low TCR and MHC affinity (24). It seems then that TLR-2-mediated stimulation of CD8^+^ T-cells decreases or even obviates the need for co-stimulation by APC improving the chances for successful CD8^+^ T-cell responses even in the presence of reduced antigen and low affinity TCR. Secondly, inflammation has been shown by many groups to play a crucial role in the contraction phase and development of memory CD8^+^ T-cell responses (25–27). The inflammatory milieu induced by the pathogen has been found to be essential for maximal CD8^+^ T-cell expansion and is also crucial for the development of effector functions such as cytolysis (28). Furthermore, Richer and colleagues (29) have shown that inflammatory cytokines reduce antigen sensitivity in both effector and memory CD8^+^ T-cell responses.

The development of prophylactic agents that augment the host’s innate immune system could considerably decrease the morbidity and mortality rates that are associated with influenza pandemics for which no vaccines are available or in those cases where available vaccines are ineffective, e.g., during the 2009 H1N1 influenza pandemic where the only seasonal influenza vaccine that was available failed to induce immune responses capable of protecting individuals against the emergent strain (30). Intranasal administration of PEG-Pam_2_Cys at such times could provide the population with immediate protection and reduce transmission of virus (1). As we show in this study, individuals treated with Pam_2_Cys and subsequently challenged with virus would develop influenza-specific adaptive immune responses providing long-term protection and removing the need for rapid vaccine production. Another feature of PEG-Pam_2_Cys as an immunostimulatory agent is that it has the potential to be self-administered reducing the impact placed on medical staff during pandemics.

We have shown that the TLR-2 agonist PEG-Pam_2_Cys provides mice with immediate protection against influenza virus and does not impact the induction of influenza-specific immunity following subsequent exposure to virus, which provides both homotypic and heterosubtypic protection. The data generated in this study encourages the development of immunostimulatory agents and could also alter our perception of the role that these anti-microbial agents play in long-term immunity to respiratory infections. In the absence of an effective vaccine, the use of Pam_2_Cys can immediately reduce the impact of infectious agents and provide an individual with long-lasting immunity through natural infection.

## Conflict of Interest Statement

The authors declare that the research was conducted in the absence of any commercial or financial relationships that could be construed as a potential conflict of interest.

## Supplementary Material

The Supplementary Material for this article can be found online at http://journal.frontiersin.org/article/10.3389/fimmu.2015.00290/abstract

## Abbreviations

Ab, antibody; Influenza, influenza A virus; Pam_2_Cys, *S*-[2,3-bis(palmitoyloxy)propyl] cysteine; TLR, toll-like receptor.
