# Supplementary material for: Generation of Adaptive Immune Responses Following Influenza Virus Challenge is Not Compromised by Pre-Treatment with the TLR-2 Agonist Pam2Cys
Source: Front Immunol. 2015 Jun 5;6:290. doi: 10.3389/fimmu.2015.00290 (PMC4457020; doi:10.3389/fimmu.2015.00290)
Supplement: Supplementary file 1 [file Presentation_1.PDF]

Figure S1 Gating strategy used to identify triple positive cells.
C57BL/6 mice (n=5) received 20nmol of PEG-Pam$_2$Cys or saline 3 days prior to challenge with Mem/Bel. An ICS was performed 10 days after influenza challenge to examine the cytokine profile of influenza specific CD8$^+$ T-cells that were generated. Lymphocytes were identified based on their size. The number of CD8 T-cells secreting IFN-γ was identified from this fraction. This cell population was further characterized based on their ability to secrete IFN-γ and TNF-α. The double positive cell fraction was used to identify the triple positive cells.

Figure S2: Gating strategy used to for *in vivo CTL* assay.
C57BL/6 mice (n=5) received saline or PEG-Pam$_2$Cys 3 days prior to challenge with $10^{4.5}$ PFU of Mem71 influenza virus. One month later mice were challenged with a lethal dose of PR8 and seven days later naïve "donor" splenic cells were differentially labeled with CFSE and pulsed with either no peptide, peptide $NP_{366-374}$ or peptide $PA_{224-236}$ before intravenous transfer via the base of tail into recipient mice. Recipient mice were killed and remaining labeled donor cells in the lungs and spleens enumerated using flow cytometry. The gating strategy used to identify CFSE labeled cells in the lungs of Naive (A) and infected mice (B) is shown above. Cells were first gated on size and then 3 distinct populations were identified based on their differential expression of CFSE and DID. The histograms represent the number of peptide pulsed cells expressing CFSE remaining in the lungs.

Figure S3. Gating strategy used to identify cytokine-secreting cells.
C57BL/6 mice (n=5) received 20nmol of PEG-Pam$_2$Cys or saline 3 days prior to challenge with Mem71. One month after primary challenge mice were challenged with PR8 influenza virus and 7 days later an ICS assay was performed to examine the cytokine profile of influenza specific CD8$^+$ T-cells that were generated. Lymphocytes were identified based on their size. The number of CD8 T-cells were identified from this fraction. This cell population was further identified based on their ability to secrete IFN-γ and TNF-α.

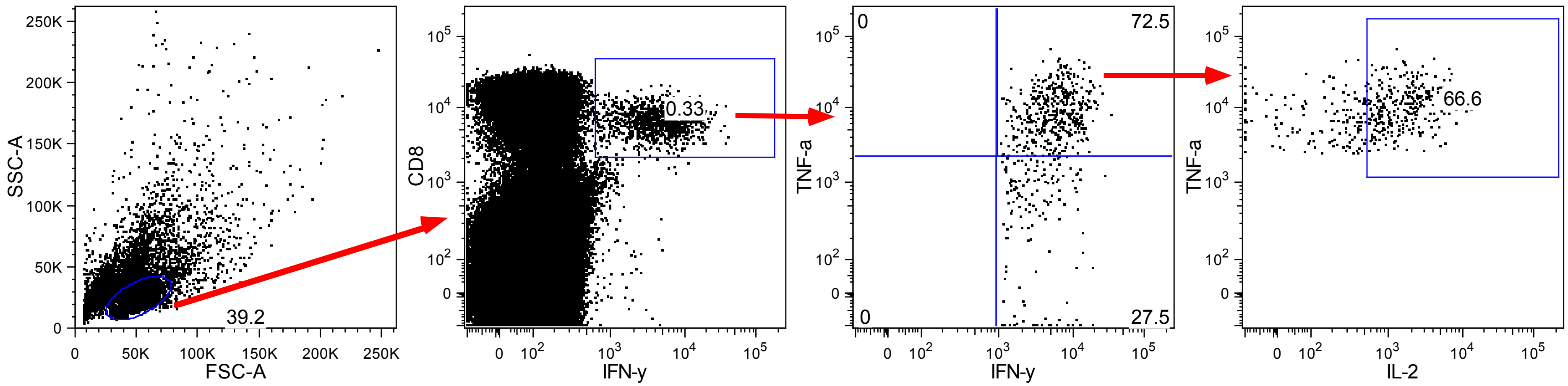

Figure S1

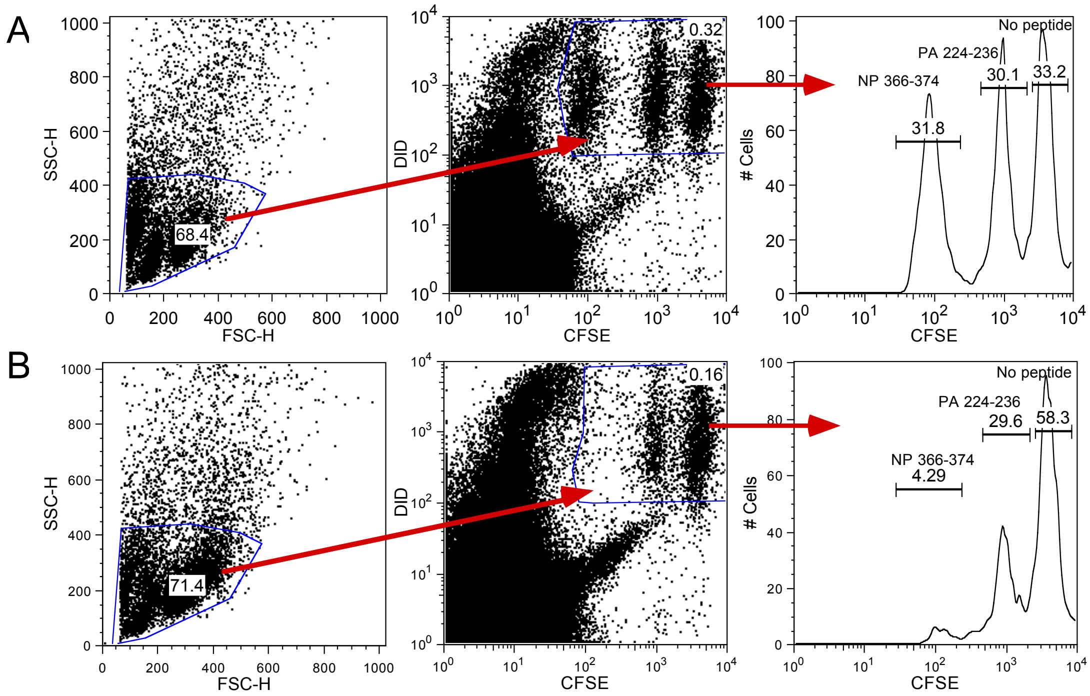
A
SSC-H
FSC-H
68.4
DID
CFSE
0.32
Cells
No peptide
PA 224-236
NP 366-374
31.8
30.1
33.2
B
SSC-H
FSC-H
71.4
DID
CFSE
0.16
Cells
No peptide
PA 224-236
29.6
58.3
NP 366-374
4.29

Figure S2

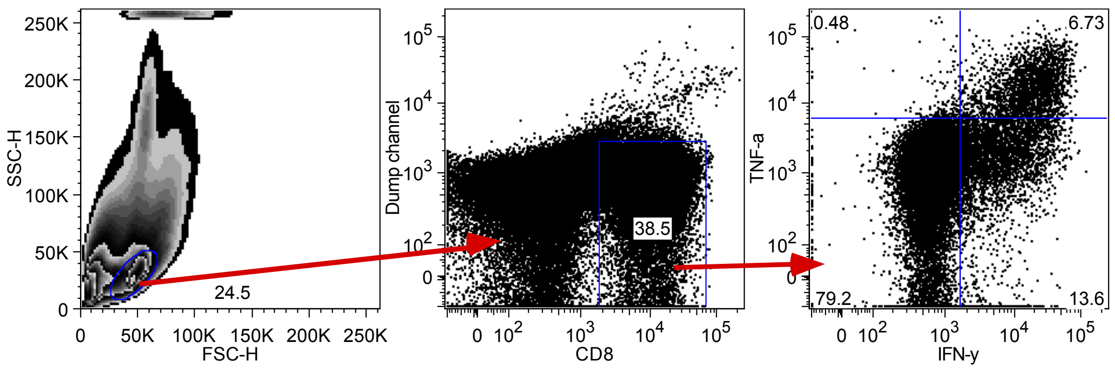

SSC-H
250K
200K
150K
100K
50K
0
FSC-H
0
50K
100K
150K
200K
250K
24.5
Dump channel
CD8
38.5
TNF-a
IFN-y
0.48
6.73
79.2
13.6

Figure S3
